# Lithium niobate on insulator – fundamental opto-electronic properties and photonic device prospects

**DOI:** 10.1515/nanoph-2024-0132

**Published:** 2024-05-29

**Authors:** Bin You, Shuangxiu Yuan, Yuan Tian, Haisu Zhang, Xiaolong Zhu, N. Asger Mortensen, Ya Cheng

**Affiliations:** State Key Laboratory of Precision Spectroscopy, School of Physics and Electronic Science, 12655 East China Normal University, Shanghai 200241, China; 6174 POLIMA—Center for Polariton-driven Light–Matter Interactions, University of Southern Denmark, Campusvej 55, DK-5230 Odense M, Odense, Denmark; Danish Institute for Advanced Study, University of Southern Denmark, Campusvej 55, DK-5230 Odense M, Odense, Denmark

**Keywords:** photonics devices, lithium niobate on insulator, nonlinear-optical effect, tunable properties, structure colours

## Abstract

Lithium niobate on insulator (LNOI) combines a variety of optoelectronic properties and can meet practical performance requirements that are uncommon in optoelectronic materials. This review introduces the fundamentals and the photonic device concepts that arise from the LNOI materials platform. Firstly, the nonlinear optical response of LNOI is presented, including birefringent phase matching (BPM), modal phase matching (MPM), and quasi-phase matching (QPM). The tunable properties are also introduced, including electro-optical (EO), thermo-optical (TO), and acousto-optical (AO) effects. The structures of nonlinear optical devices, such as ridge waveguides (including periodically polarized inversion waveguides), Mach–Zehnder interferometer (MZI) modulators and micro-resonators (such as disks and rings) are demonstrated. Finally, the future of LNOI devices is discussed. In the already mature and developed optoelectronic material systems, it is rare to find one particular material system supporting so many basic optical components, photonic devices and optoelectronic devices as LNOI does in the field of integrated photonic chips.

## 1 Introduction

For decades, as photonics concepts have become more integrated into optoelectronic devices, there has been a wide range of researches on suitable materials to modulate optical signals [1]–[8]. As the new technology has been driven by the growing demand for the Internet of Things (IoT) and big data connections, optical devices are required to provide a large bandwidth, small footprint, low optical loss, low energy consumption, and high integration [9]–[14]. The selected silicon (Si) and group III–V semiconductors have encountered growing challenges in meeting the demands of modern cutting-edge optics. Scientists have been searching for suitable materials with low power consumption, high speed, and high efficiency to generate and manipulate optical signals.

**Figure 1:**
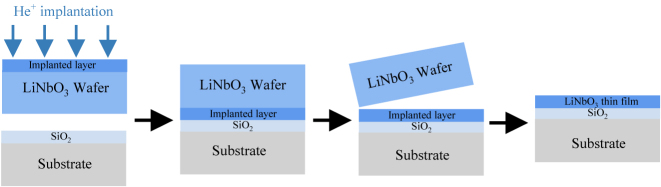
Schematic preparation of LNOI by ion slice.

LNOI, referred to as the silicon of photonics [15]–[17], has the advantages of small device footprints, high bandwidth, and low loss. The key material properties of LN are compared to those of other popular materials used in integrated photonics, as shown in the reference. [18]. Compared to semiconductors such as silicon (Si) [19]–[26] and indium phosphide (InP) [27]–[29], polymers [30]–[32], plasmonic metals [33]–[35], and phase-change materials [36]–[39], LNOI exhibits good nonlinearity and high extinction ratio in the infrared communication band [40]. In the last few years, with the rapid development of LNOI technology and significant advances in related surface structures and engineering technologies, “Ion Slicing” has enabled the production of large-scale, high-quality (*Q*-factor), and sub-micrometre-thick crystalline LN thin films. As illustrated in Figure 1, He^+^ ions are first implanted into the submicron single crystal LN film, which determines the final thickness of the LN thin film. Many new chip-integrated devices and applications based on LNOI have been demonstrated with low cost/power consumption/complexity, ease of processing, and small size. This has driven the resurgence of integrated photonics using this material [41]–[46]. As a well-known multifunctional material, LNOI has been widely used in photonics, electronics, and optoelectronics for its birefringence effect, nonlinear effect, EO effect, TO effect, and AO effect [47]–[52]. LNOI has gained great attention from experimental and theoretical scientists, achieving many promising applications such as second harmonic generation (SHG), photonics chips, wavefront shaping, and structural colors [53]–[55].

In this paper, we provide a review of the physical mechanisms of LNOI devices, the design strategies for components, the practical implementations, and the emerging applications. Firstly, we explore the nonlinear effect, one of the most attractive features of LNOI. In this case, we will use three-wave mixing as an example, and we will introduce two methods for generating the nonlinear effect, such as BPM, MPM, and QPM. We will also present the linear EO effect, linear TO effect, and AO effect, which are widely used modulation methods in LNOI. Next, a series of structures of nonlinear photonic devices based on the LNOI are introduced, such as ridge waveguides, periodically poled inversion waveguides, MZI modulators, and micro-resonators like disks and rings. Furthermore, the concepts of metasurfaces and photonic crystals are introduced due to their numerous applications, such as SHG, wavefront shaping, and topological control. Finally, conclusions and prospects are presented. Here, the future direction of LNOI devices and their development prospects are briefly analysed. We believe that this review would offer an interesting introduction of LNOI, providing both in-depth discussions and perspectives.

### 1.1 Nonlinear optical effect of LNOI

Shortly after the invention of the ruby laser in 1961, Franken et al. used a ruby laser with a 694 nm output wavelength to produce 347 nm ultraviolet light when light passing through a quartz crystal. This is considered the first observed optical SHG phenomenon, marking the birth of nonlinear optics. With the development of nonlinear optics, its applications are becoming increasingly widespread in scientific research [56], [57]. One of the most important research aspects is that lasers of different frequencies can exchange energy within a nonlinear crystal. This exchange causes electron energy level transitions within the medium, leading to the generation of light waves with new frequencies [58], [59]. The SHG is related to the second-order polarization in the nonlinear polarization term. Similarly, other nonlinear phenomena such as the third harmonic, higher harmonics, sum and difference frequencies, optical parametric generation, optical parametric amplification, oscillation, and spontaneous parametric down-conversion are also associated with the relevant nonlinear polarization terms [60], [61]. These optical frequency conversion effects can yield highly efficient coherent light output and enable a wide range of tunable light sources. For example, it can upconvert single-photon signals in the infrared (IR) wavelengths to the visible wavelengths [62]–[66]. There are three main theoretical frameworks in nonlinear optics: the classical, semi-classical, and full quantum approaches to solve the interaction between light and matter. In classical theory, the light field is described as a classical electromagnetic field within Maxwell’s theory, and the medium is composed of classical oscillators, conceptually governed by classical mechanics. The semi-classical treatment involves a classical electromagnetic field within Maxwell’s theory, while the medium consists of particles with quantum properties described by quantum mechanics. Finally, in the full quantum theory, the otherwise classical light field is quantized, which we know as quantum optics. In this paper, we will introduce the classical theory and its semi-classical extension, which is a framework that has been able to account for most of the nonlinear-optics problems in practical applications. With the rapid development of nonlinear optics, the understanding of nonlinear optics is gradually shifting from initially being fundamental phenomena and principles of light–matter interactions to the broad real-life applications that we see today [67]–[71].

The pioneering theoretical work on nonlinear optics, which constitutes the essence of nonlinear optics and forms the basis for light–matter interactions and the concept of “controlling light with light”, was carried out by Bloembergen and others in the 1960′ties [72]. Here, we take frequency doubling as an example. The fundamental light and the frequency-doubled light propagate together when the frequency-doubled light is excited by the fundamental light. Determined by its point group of 3 m, the nonlinear coefficient tensor *d*_*ij*_ of LN can be expressed as:

(1) $$\begin{array}{c} d_{ij}=\left[\begin{array}{cccccc} 0 & 0 & 0 & 0 & d_{31} & -d_{22}\\ -d_{22} & d_{22} & 0 & 0 & 0 & 0\\ d_{31} & d_{31} & d_{33} & 0 & 0 & 0 \end{array}\right], \end{array}$$

where the second-order nonlinear coefficients are *d*_33_ = 27 pm/V, *d*_31_ = 4.6 pm/V, and *d*_22_ = 3 pm/V [18]. Therefore, when a strong fundamental wave (FW) pumped, the second-harmonic (SH) polarizability strength $P_{i}\left(2\omega \right)\;\;\left(i=x,y,z\right)$ formed inside the LN would be:

(2) $$\begin{array}{ll} \left[\begin{array}{c} P_{x}\left(2\omega \right)\\ P_{y}\left(2\omega \right)\\ P_{z}\left(2\omega \right) \end{array}\right] & =\left[\begin{array}{cccccc} 0 & 0 & 0 & 0 & d_{31} & -d_{22}\\ -d_{22} & d_{22} & 0 & 0 & 0 & 0\\ d_{31} & d_{31} & d_{33} & 0 & 0 & 0 \end{array}\right]\\ & \;\times \left[\begin{array}{c} E_{x}^{2}\left(\omega \right)\\ E_{y}^{2}\left(\omega \right)\\ E_{z}^{2}\left(\omega \right)\\ 2E_{y}\left(\omega \right)E_{z}\left(\omega \right)\\ 2E_{x}\left(\omega \right)E_{z}\left(\omega \right)\\ 2E_{x}\left(\omega \right)E_{y}\left(\omega \right) \end{array}\right], \end{array}$$

where the $E_{i}\left(\omega \right)\;\left(i=x,y,z\right)$ is the electric field of FW and $P_{i}\left(2\omega \right)\;\left(i=x,y,z\right)$ is the electric field of SH in *x*/*y*/*z* component.

However, due to the frequency dispersion of the host material, the fundamental and doubled frequency light travel at different speeds. The waves of fundamental light and the frequency-doubled light become spatially misaligned after a certain distance. Simultaneously, the fundamental light still produces a constant stream of the new frequency-doubled light. The new frequency-doubled light is coherent with the original frequency-doubled light and potentially cancels it out when the fundamental light and the frequency-doubled light pulses are half a wavelength apart due to the dispersion. Therefore, we can align the phase of the newly generated frequency-doubled light with that of the previously generated frequency-doubled light.

**Figure 2:**
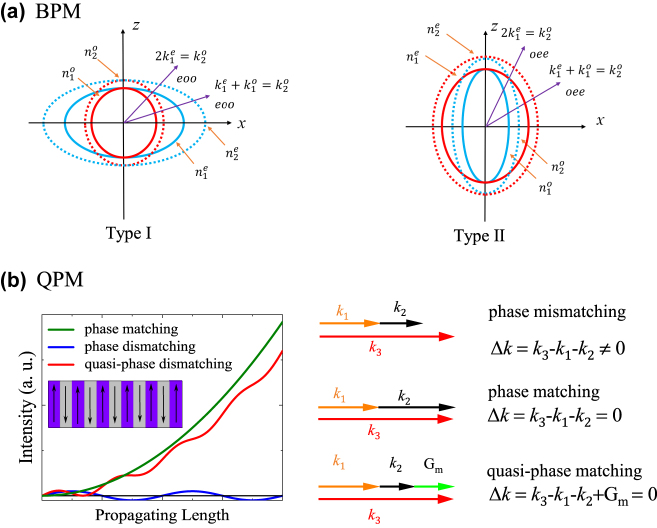
Diagram of (a) birefringent phase matching and (b) intensity of harmonic wave with respect to interaction distance of phase mismatching, phase matching and quasi-phase matching cases [72], [74].

For instance, in the three-wave mixing [73], three waves are characterized by the amplitudes *A*_*i*_ and the wave vectors *k*_*i*_, where *i* = 1, 2 is for the incident light, *i* = 3 denotes the outgoing light and Δ*k* is phase (momentum) mismatch given by Δ*k* = *k*_1_ + *k*_2_ − *k*_3_. Obviously, the amount of phase (momentum) mismatch is a key factor affecting the frequency conversion efficiency. The other higher-order nonlinear responses also follow perfect phase matching (PM) [74], when particular situation with Δ*k* = 0 and e^iΔ*kz*^ = 1 (see Figure 2) happens. However, nonlinear crystals typically have wave vectors that deviate slightly from the perfect matching situation, causing the signal to decay after a coherence length. Therefore, BPM and QPM are introduced in the following.

#### 1.1.1 Birefringent phase matching

BPM exploits the different propagation speeds (refractive indices) of polarized light in birefringent crystals for phase matching. This method involves selecting a particular polarization direction and propagation direction for the fundamental frequency light and multifrequency light to ensure that the beams propagate in the crystal along the phase-matched specific direction [75], [76]. In general, birefringent crystal materials are widely used in nonlinear optics, such as barium metaborate (BBO) [77], potassium dihydrogen phosphate crystal (KDP) [78], lithium niobate (LN) [79]–[81], etc.

The orientation and shape of the refractive index ellipsoid are subject to constraints imposed by the underlying symmetry of the crystal [82]. In practice, the crystals are cut along a specific direction to ensure that the beams meet at the phase-matched angle inside the crystals upon incidence, thereby achieving the effect of phase matching. There are two options for the selection of polarization: type I phase matching and type II phase matching. In type I phase matching, the polarization states of the fundamental-frequency light are the same, while in type II phase matching, they are different. The birefringent phase matching diagrams of positive and negative uniaxial crystals are shown in Figure 2(a).

However, there are also disadvantages, such as the short-wave limit and walk-off effect. BPM has some drawbacks of its own. A specific phase match determines the individual wave polarization directions and the BPM angles. As a result, the maximum tensor element of the nonlinear coefficients cannot be fully utilized, leading to low conversion efficiency. Moreover, BPM is rarely implemented in waveguides. So far, achieving the BPM SHG approach at 1064 nm has mainly relied on temperature tuning [83]–[85], where the conditions for BPM are naturally satisfied. Perfect BPM synchronization in the telecommunication band is rarely achieved in LNOI waveguides. From a group-theory perspective, the LN is the 3 m point group of the tripartite crystal system with *n* = 3. The refractive index ellipsoid is a rotating ellipsoid with *z* as the symmetry axis. In 1997, Zelmon et al. demonstrated the first experiment [86].

#### 1.1.2 Modal-phase matching

MPM is a straightforward solution to the issue of phase velocity synchronism in nonlinear frequency conversion processes. It is well-known but has garnered limited interest due to the inadequate spatial overlap between the interacting modes. For most LNOI devices, the angle of the waveguide is determined during the fabrication process. Nonlinear LNOI devices primarily rely on the spatial eigenmodes of the waveguides instead of the bulk refractive index. The concept can be considered as modal phase matching instead of BPM [75]. More importantly, the size of the cross-section of the waveguide is on the wavelength scale, so the structure induces a much larger dispersion of the eigenmode index than the material dispersion. Indeed, the examples mentioned in the BPM section also depend on the high-order spatial modes at the pump frequency [79], [80]. While the type-0 phase matching condition (where all frequency components have the same polarization states) cannot be achieved in BPM, it can be readily achieved with LNOI devices using the concept of the MPM. By carefully designing the waveguide geometries and engineering its dispersion, MPM between the fundamental mode and a higher-order mode could be achieved, enhancing the conversion efficiency significantly. For example, the phase-matching condition can be satisfied at any azimuthal angle in Z-cut LN films that are isotropic in the device plane. Therefore, the strong SHG is expected to occur as the fundamental-frequency (FF) light travels around the cavity [87].

#### 1.1.3 Quasi-phase matching

QPM, proposed by Bloembergen in 1962 [72], is an alternative method to compensate for the phase mismatch between fundamental-frequency waves and higher harmonics during propagation in nonlinear materials. This method involves the periodic modulation of the nonlinear polarization rate of the crystal [88], [89].

As seen in Figure 2(b), the harmonic energy oscillates periodically with the distance between the fundamental-frequency light and the nonlinear crystal, affecting the phase mismatch. At this point, the coherence length (*L*) is defined as the distance after half a period of oscillation. We find that the light intensity energy of the multiplied light reaches the maximum and then decreases within the first coherence length. Then, the newly generated second harmonic and the previously generated second harmonic components are in opposite phases within the second coherence length. As a result, the coherence phase vanishes, and the energy of the light reverts back to the fundamental-frequency light. In the first distance of *L*, the phase difference of the light wave transmission accumulates from 0 to π, resulting in constructive interference, and the harmonic intensity gradually increases. However, at a distance of 2*L*, the phase accumulates from π to 2π, leading to destructive interference, and the harmonic intensity gradually decreases. Periodically reversing the domain structure results in a change in the sign of the crystal’s second-order polarizability, leading to a continuous enhancement of the harmonic intensity. LN, as a ferroelectric crystal, has many small regions or domains, and the spontaneous polarization inside each small domain is along the same direction. The direction of spontaneous polarization in the crystal changes by 180° after the inversion of the periodically polarized LN domains. The nonlinear coefficients are discontinuous at the domain walls, and the second-order nonlinear coefficients change in the laboratory coordinate system. The nonlinear coefficients are inverted in the extremely narrow regions on both sides of the domain walls.

Dispersion engineering is a critical aspect that can greatly enhance nonlinear optical processes with QPM. QPM can be achieved for almost any waveguide geometry of interest, which liberates the geometric dispersion as a design parameter. This freedom enables a new set of design rules where multiple dispersion orders, such as the group velocities and group-velocity dispersion of the interacting waves, can be simultaneously engineered to achieve favorable characteristics across a wide range of wavelengths [13]–[17], [80]. The design rules have been provided for dispersion-engineered QPM devices, with a particular focus on how to engineer the bandwidths of nonlinear interactions by Fejer et al. [90]. These rules are applied to the design of nonlinear components that can be utilized to generate and manipulate quantum light.

### 1.2 Tunable properties

Tunable materials enable the electromagnetic properties to be altered by applying an external signal. This can, on the one hand, change and extend the operating range of the material and, on the other hand, offer the possibility of developing various active devices such as modulators. There are currently three main types of mechanisms for tunable materials: firstly, circuit-based approaches, where semiconductor materials are inserted into the microstructure unit that can change the impedance of the circuit [91]. Secondly, geometry-based approaches involve physically altering the geometry of the microstructure to change its equivalent parameters [92]. And finally, material property-based approaches involve constituent material microstructure units that are composed of tunable materials [93]. In terms of the characteristics of the optical signals being modulated, there are amplitude modulation, resonant frequency modulation, phase modulation, etc. The highly tunable properties of LN crystals are caused by the lattice structure and the rich defect structure. The properties of materials can often be significantly adjusted through electrical, thermal, and acoustical modulation methods.

#### 1.2.1 Electro-optical effect

After applying an electric field to the crystal, the shape, size, and orientation of the refractive index ellipsoid will change. The cross term in the refractive index is caused by the electric field, indicating that the deformed ellipsoidal principal axes do not coincide with the original ones. The relationship between the refractive index and the electric field can be expressed as:

(3) $$\left\{\begin{array}{c} \frac{1}{n_{11}^{2}}-\frac{1}{n_{1}^{2}}=\gamma_{11}E_{x}+\gamma_{12}E_{y}+\gamma_{13}E_{z}\;\\ \frac{1}{n_{21}^{2}}-\frac{1}{n_{2}^{2}}=\gamma_{21}E_{x}+\gamma_{22}E_{y}+\gamma_{23}E_{z}\;\\ \frac{1}{n_{31}^{2}}-\frac{1}{n_{3}^{2}}=\gamma_{31}E_{x}+\gamma_{32}E_{y}+\gamma_{33}E_{z}\;\\ \frac{1}{n_{23}^{2}}=\gamma_{41}E_{x}+\gamma_{42}E_{y}+\gamma_{43}E_{z}\;\\ \frac{1}{n_{23}^{2}}=\gamma_{51}E_{x}+\gamma_{52}E_{y}+\gamma_{53}E_{z}\;\\ \frac{1}{n_{23}^{2}}=\gamma_{61}E_{x}+\gamma_{62}E_{y}+\gamma_{63}E_{z}\; \end{array}\right.,$$

where *n*_*ij*_ (*i*, *j* = 1, 2, 3) is the refractive index of the ellipsoid and *γ*_*ij*_ (*i* = 1, 2, 3 … 6, *j* = 1, 2, 3) is the EO coefficient. As a crystal system belonging to the tripartite crystal system and the 3 m point group, the refractive index sphere of LN is a rotating ellipsoid with the axis of symmetry, and the cross-section perpendicular to the axis is a circle. Its electro-optical coefficients are *γ*_13_ = *γ*_23_, *γ*_22_ = −*γ*_12_ = −*γ*_61_, *γ*_42_ = *γ*_51_, while the rest are zero. The induced principal refractive index of the induction spindle (*n*_*x*_, *n*_*y*_, *n*_*z*_) is:

(4) $$\left\{\begin{array}{c} n_{x}≅n_{o}+\frac{1}{2}n_{o}^{3}\gamma_{22}E\;\\ n_{y}≅n_{o}-\frac{1}{2}n_{o}^{3}\gamma_{22}E\;\\ n_{z}=n_{e}\; \end{array}\right.$$

**Figure 3:**
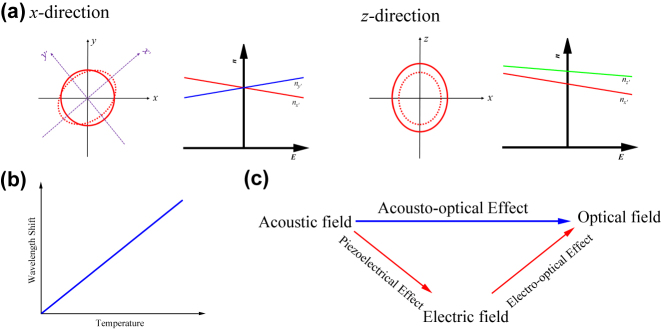
Tunable properties of LN. (a) The refractive index ellipsoid when add the electric field from *x/y* or *z* direction and their variation in refractive index of principal axes is linear. (b) The wavelength shift of LN varies linearly with temperature. (c) The principle of LN acousto-optical effect is that the periodic pressure of sound waves produces an electric field under the effect of piezoelectricity, and the electric field leads to a change in refractive index which changes the optical field [14].

As is shown in Figure 3(a) and Eq. (4), after adding the *x*/*y* electric field component, the three principal directions rotate by 45°. The original cross-section changes from a circle to an ellipse: the *y*/*x* axis shortens, and the *x*/*y* axis elongates. The length of the induction axis is, in a first approximation, linearly related to the applied electric field.

As for addition of an electric field in the *z* direction, the induced principal refractive index of the induction spindle (*n*_*x*_, *n*_*y*_, *n*_*z*_) is:

(5) $$\left\{\begin{array}{c} n_{x}≅n_{o}-\frac{1}{2}n_{o}^{3}\gamma_{13}E\;\\ n_{y}≅n_{o}-\frac{1}{2}n_{o}^{3}\gamma_{13}E\;\\ n_{z}≅n_{e}+\frac{1}{2}n_{e}^{3}\gamma_{33}E\; \end{array}\right.\cdot$$

As seen in Figure 3(a) and Eq. (5), after adding the *z* electric field component, the ellipsoid deforms: the *x* and *y* axis all shorten, while the *z* axis elongates. Also, the length of the induction axis is linearly related to the applied electric field in a first approximation.

#### 1.2.2 Thermo-optical effect

Like the EO effect, the TO effect is the phenomenon where the refractive index of a material changes with temperature, depending on the crystal axis of LN. The TO coefficient is the ratio of the change in the refractive index of an optical material to the temperature. This ratio typically differs among various optical materials. Schlarb and Betzler derived a generalized Sellmeier formula that considers all the factors and parameters affecting the refractive index value, including wavelength, composition, and temperature, on the optical properties of LN [94]. A theoretical analysis based on the thermal expansion coefficient, the TO coefficient of the excitonic bandgap, an isotropic bandgap of the TO coefficient, and its dependence on the wavelength and temperature was provided by Ghosh in 1998 [95]. In 2005, Moretti compared two theoretical models and experimental data on the temperature dependence of the TO coefficient of LN from 300 to 515 K in the visible and infrared regions [96]. Later, Fieberg measured the thermal expansion coefficient of LN in the temperature range from 283 to 433 K using interferometry and a Schott equation to describe the wavelength and temperature dependence of the thermal expansion coefficient [97]. Noted that the high TO coefficient of the LN enables the implementation of linearly tunable devices through temperature (see Figure 3(b)) [98]–[102].

#### 1.2.3 Acousto-optical effect

There is a long tradition of studying the interaction between light and mechanical waves, which is widely utilized to synthesize various AO devices used in optical networks and signal processing applications [103], [104]. Thin film AO devices have facilitated the advancement of piezoelectric transducer technology and the rapid expansion of integrated optics. As seen in Figure 3(c), the fundamental of an AO modulator in LNOI is the piezo-optomechanical (PO) effect based on the high electromechanical coupling [105], which converts signals among optical, acoustic, and radio waves [106]. Piezoelectricity is the phenomenon in which an electric field is generated by an elastic wave (or vice versa), playing a crucial role in the overall coupling from microwaves to light. The optomechanical approach with gigahertz (GHz) mechanical devices has the potential to be extremely efficient due to the large piezoelectric response of LN and the ability to localize mechanical energy into a micron-scale volume. The photo-elastic coefficient *P* can be given by

(6) $$\begin{array}{c} P=\left[\begin{array}{cccccc} p_{11} & p_{12} & p_{13} & p_{14} & 0 & 0\\ p_{12} & p_{11} & p_{13} & -p_{14} & 0 & 0\\ p_{31} & p_{31} & p_{33} & 0 & 0 & 0\\ p_{41} & -p_{41} & 0 & p_{44} & 0 & 0\\ 0 & 0 & 0 & 0 & p_{44} & p_{41}\\ 0 & 0 & 0 & 0 & p_{14} & p_{66} \end{array}\right]. \end{array}$$

The photo-elastic coefficient tensors *p*_*ij*_ have been experimentally measured [15]. The variation of refractive index can be express as:

(7) $$\begin{array}{c} \Delta n_{i}=-\frac{1}{2}n_{i0}^{3}\underset{j}{\sum }p_{ij}\cdot S_{j}, \end{array}$$

where ***n***_***i*** 0_ is the original refractive index of *x*/*y* component and ***S***_***j***_ is the strain field. By channeling phonons into the optomechanical device, the phonons could alter the properties of the light trapped in the AO device, enabling the manipulation of the motion of the nanoscale beam [107].

## 2 Optical devices based on LNOI

The refractive index of LN is *n*_*o*_ = 2.21 and *n*_*e*_ = 2.14 [86]. Compared to Silicon with *n* = 3.48 [108], LN has a lower refractive index. This naturally leads to larger device footprints compared to Si device platform, which not only reduces the integration density of optoelectronic chips but also places higher demands on computational resources during the design phase and in the fabrication processing time. Therefore, fabricating optical microstructures on thin-film lithium niobate platforms to reduce device size and increase chip integration is a very attractive option. So far, a few applications of LNOI optics, such as SHG, EO, AO, TO, wavefront shaping, and Kerr optical OFC, have been extensively developed [109]–[115]. Here, we report on several structures commonly utilized to achieve linear or nonlinear optical effects, such as waveguides, MZI structures, and micro-resonators (disks and rings).

### 2.1 Ridge waveguide

**Figure 4:**
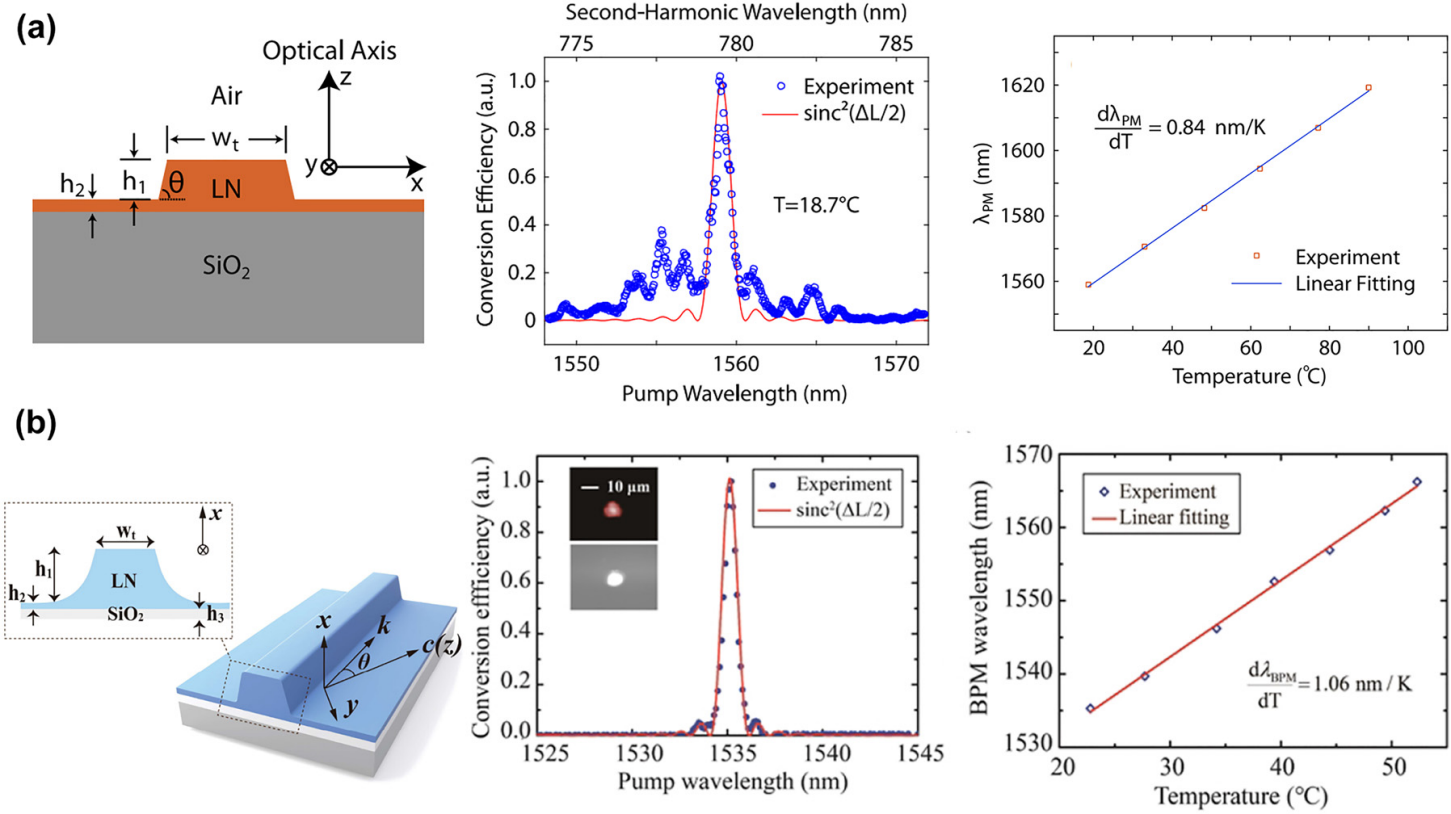
A novel design of angle-cut ridge waveguides for SHG were propose and demonstrate on LNOI platform via type-I BPM. The fundamental wave (FW) and second-harmonic (SH) waves could be generated in the ridge waveguide by carefully designing in (a) [79] and (b) [80]. The wavelength shift is linearly related to temperature and the slop is 0.84 nm/K [79] and 1.06 nm/K [80], respectively.

When the crystal lattice temperature is appropriate, the birefringence effect exactly cancels the dispersion effect, thus achieving BPM [116]. As shown in Figure 4(a), on-chip SHG was proposed and demonstrated in the ridge waveguide by Lin et al., achieving both large tunability and high conversion efficiency [79]. This LNOI nanophotonic waveguide is only 8 mm long and demonstrates highly efficient on-chip wavelength conversion. It has a tuning slope of 0.84 nm/K for a telecom-band pump and a nonlinear conversion efficiency of 4.7 % W^−1^. Recently, Chen et al. reported a ridge waveguide for type-I BPM SHG on the LNOI platform with a normalized BPM conversion efficiency of 2.7 % W^−1^ cm^−2^ in an LNOI waveguide and a tuning slope of 1.06 nm/K at the telecommunication C band [80], (see Figure 4(b)). Also, Chen et al. achieved broadband SHG of a femtosecond laser in the telecom C-band and a temperature gradient scheme [81]. The BPM may open a new pathway for tunable nonlinear frequency conversion in various integrated photonics platforms. The devices have shown great promise and have been extended to frequency conversion in integrated photonics for various applications. It should be mentioned that the BPM wavelengths were determined by the propagation or cut angle.

The second harmonic energy can grow continuously by varying the sign of the polarization rate periodically [117]. By adding electric fields or electron beams [100], [118], [119], heat treatment [120], polarization effects associated with chemical treatments [121], external diffusion of Li_2_O [122], surface chemical treatment [123], and other methods [124], [125], respectively, the periodically polarized inversion structures can be achieved directly during or after the crystal growth [126]. The realization of periodically polarized ferroelectric domain reversal structures eventually becomes a promising way to achieve QPM. Of course, there are other nonlinear crystals that can also achieve domain inversion through electric field polarization, such as LiTaO_3_ [127], [128], KTP [129], [130], etc. Experimental and application studies of QPM nonlinear frequency conversion based on this structure have been reported [41], [131], [132].

**Figure 5:**
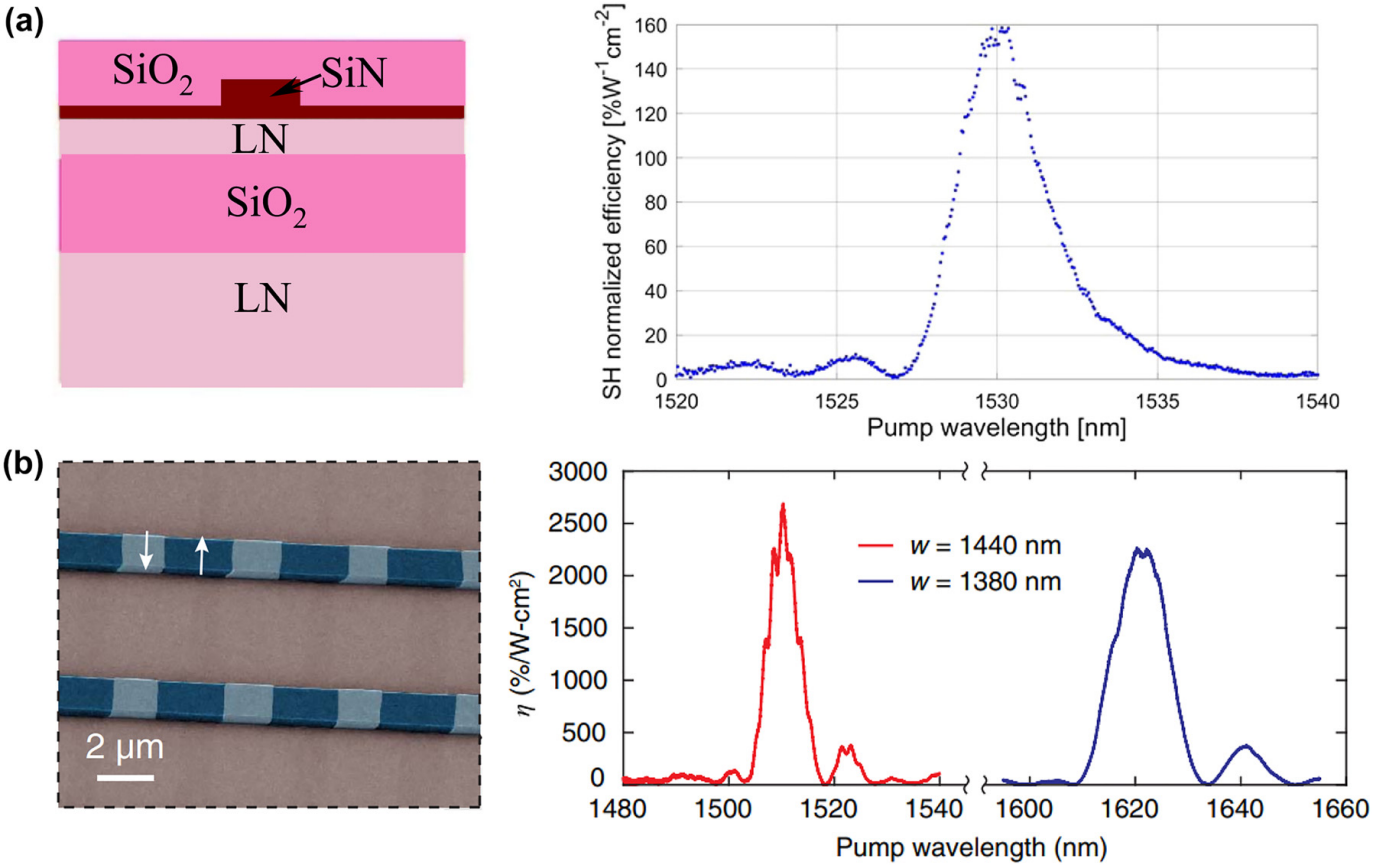
QPM was applied directly to a periodically poled LNOI waveguide. (a) The normalized SHG efficiency of 160 % W^−1^ cm^−2^ at 1530 nm with ultralow propagation loss only 0.3 dB/cm in the telecom band [134]. (b) A powerful platform for efficient wavelength conversion (2600 %/W-cm^2^) were realized at the wavelength of 1.5 μm [135].

Already in 1986, the first QPM interaction was established in the lithium niobate (LN) crystal by Min et al. [124]. They utilized spatially modulated chemical diffusion and crystal growth to generate periodic inversion of ferroelectric domains and periodic changes in the sign of the nonlinear coefficients. Later, the electric field polarization method at room temperature for ferroelectric domain inversion was developed as another approach to implementing the QPM technique [133]. After this, the QPM technique was greatly developed. Bowers and his colleagues reported a thin film wavelength converter for photonic integrated circuits [134] (see Figure 5(a)). The normalized SHG efficiency is 160 % W^−1^ cm^−2^ at 1530 nm with an ultralow propagation loss of only 0.3 dB/cm in the telecom band. More recently, Lončar et al. used a nanostructured periodically poled LNOI waveguide to achieve an ultrahigh normalized efficiency of 2600 %/W-cm^2^ for second-harmonic generation of 1500 nm radiation, which is more than 20 times higher than that in state-of-the-art diffused waveguides [135] (see Figure 5(b)).

### 2.2 Mach–Zehnder interferometer modulator

The MZI modulator primarily comprises two directional couplers and a variable phase shifter. Its functionality is based on the interference of two coherent monochromatic lights transmitted through different optical ranges. The MZI has excellent characteristics that are compatible with CMOS technology and is highly regarded for future optical computing chips [136]. So far, the MZI-type devices have been crucial components in the development of various optoelectronic devices, including EO modulators [137]–[140], AO modulators [141]–[145], Kerr optical comb [146]–[148], and more.

**Figure 6:**
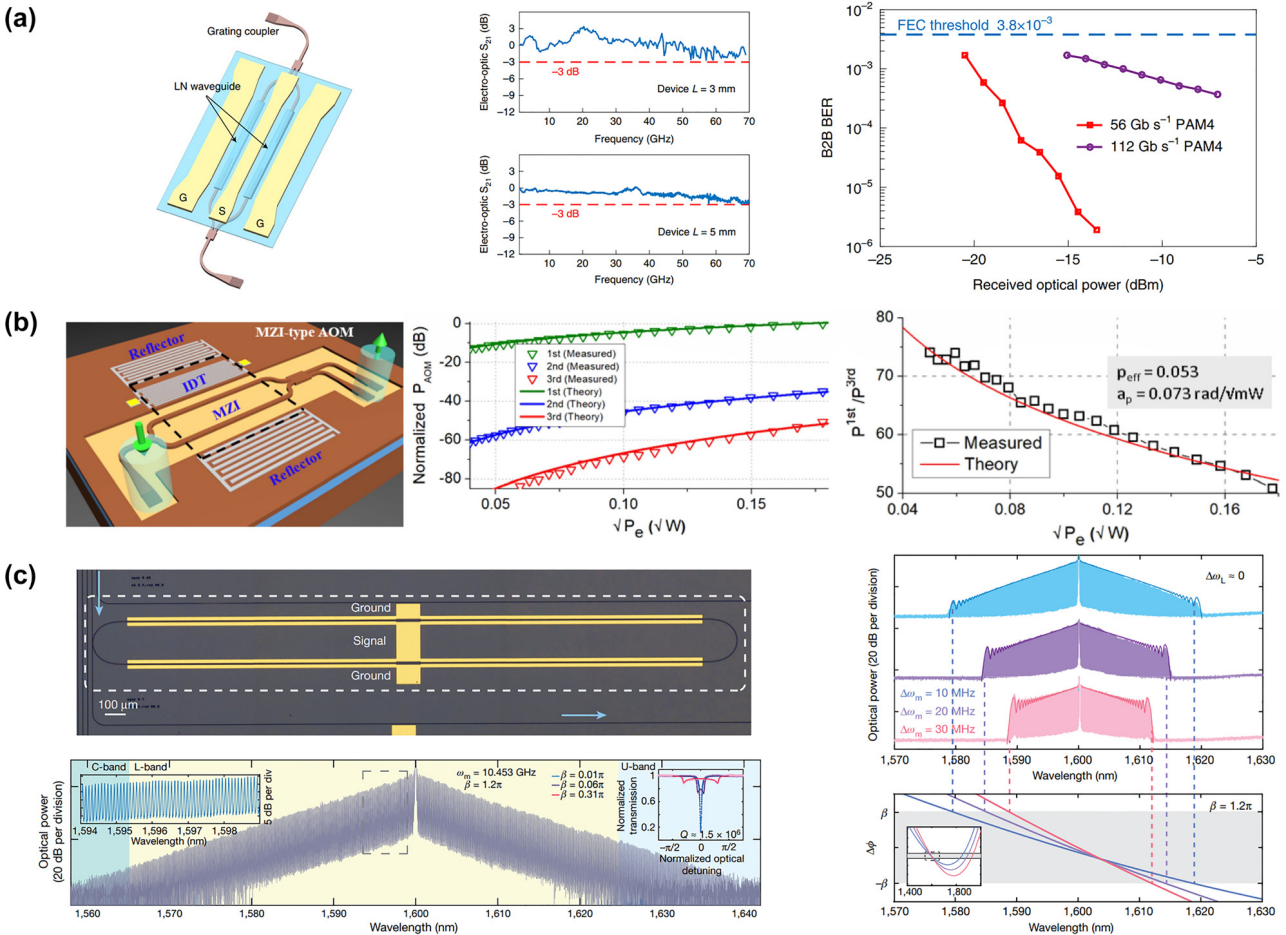
The MZI modulator based on LNOI. (a) The bandwidth and insertion loss EO modulator have reached more than 70 GHz and less than 2.5 dB [140]. (b) The high Q-factor is achieved in MZI. The normalized to the maximum first harmonic modulation power are −34 dBm. The SAW induced phase change determined the ratio of the first and third modulation harmonics (*P*_1st_/*P*_3rd_), and the experimental data fits well with the theory [141]. (c) Integrated EO OFC with a bandwidth exceeding 80 nm and more than 900 comb lines which has a large response, ultralow optical loss and highly colocalized microwave, optical fields and enabling dispersion engineering [146].

Cai et al. have designed an EO MZI modulator with hybrid integration of lithium niobate (LN) thin film and a silicon-based chip, achieving a modulation bandwidth that far exceeds that of conventional pure silicon EO modulators (>70 GHz) [140]. Record low insertion loss (<2.5 dB), modulation efficiency more than four times higher than that of conventional LNOI modulators (2.2 V cm), and excellent characteristics such as high linearity, high integration, and low cost are also presented (Figure 6(a)). In addition, the processing methods are compatible with standard CMOS process backends. Piazza et al. proposed the first acousto-optical modulator waveguide device integrated on a thin film of LNOI (500 nm), which includes surface acoustic wave generation and a photonic cavity [141]. The high optical sensitivity resonator (*Q* > 300,000) is achieved by modulating the amplitude from an on-chip MZI through the proper arrangement of the propagation directions of surface acoustic waves and optical guided modes (refer to Figure 6(b)). At the same time, a freestanding 100 μm-long thin-film acoustic resonator is reported to modulate photonics in a MZI with a half-wave voltage of *Vπ* = 4.6 V and *Vπ* = 0.77 V [142]. The acoustic resonators cause a redshift of the optical resonance as the input microwave power increases. Subsequently, the LNOI-based AO modulator developed rapidly.

In 2020, Loncar et al. experimentally demonstrated an integrated 3-GHz AO frequency shifter based on thin-film LNOI with over 30 dB carrier suppression [143]. Furthermore, the shifter can generate a gigahertz-spaced optical frequency comb with over 200 lines across a 0.6-THz optical bandwidth by circulating the light in an active frequency-shifting loop. In 2021, Yu and Sun et al. achieved amplitude modulation of gigahertz single-sideband with etch-less LN [144]. A 3 GHz frequency shifter was achieved in the C-band with a 3 dB bandwidth of approximately 35 nm. In 2022, Li et al. reported a non-suspended thin-film LNOI MZI waveguide with a low half-wave-voltage-length (<0.03 V cm) EO modulator based on built-in push-pull, which could be compared to that of a state-of-the-art suspended counterpart [145]. Besides, MZI LN-based OFC, usually combined with EO modulation, has been extensively studied by the Lončar group. In 2019, they developed an integrated EO comb generator with a significant EO response, extremely low optical loss, and highly stable microwave and optical fields, enabling dispersion engineering in a LNOI (see Figure 6(c)) [146]. In 2022, they proposed an OFC-based femtosecond pulse source using a LNOI time-lens with cascaded low-loss EO amplitude and phase modulators and chirped Bragg gratings [147].

### 2.3 Micro-resonator (disk, ring)

The on-chip LNOI micro-resonator, which includes disk or ring structures, has attracted attention due to its nonlinear optical effect [148]–[150]. In 2015, Xu and his colleagues fabricated LNOI wafers using UV lithography, reactive ion etching, and hydrogen fluoride etching [151], [152]. Likewise, Cheng et al. developed a new method for processing LNOI with significantly increasing *Q*-factors by using femtosecond laser direct writing and chemo-mechanical polishing (CMP) processes. This advancement represents a significant progress in the fabrication of high-quality LNOI micro-resonators [153]–[169]. With the advancement of processing technology, many nonlinear optical effects have also been realized using ring resonators [170]–[173]. Since then, experimental studies of nonlinear effects in LNOI micro-resonators have been reported [174], [175].

**Figure 7:**
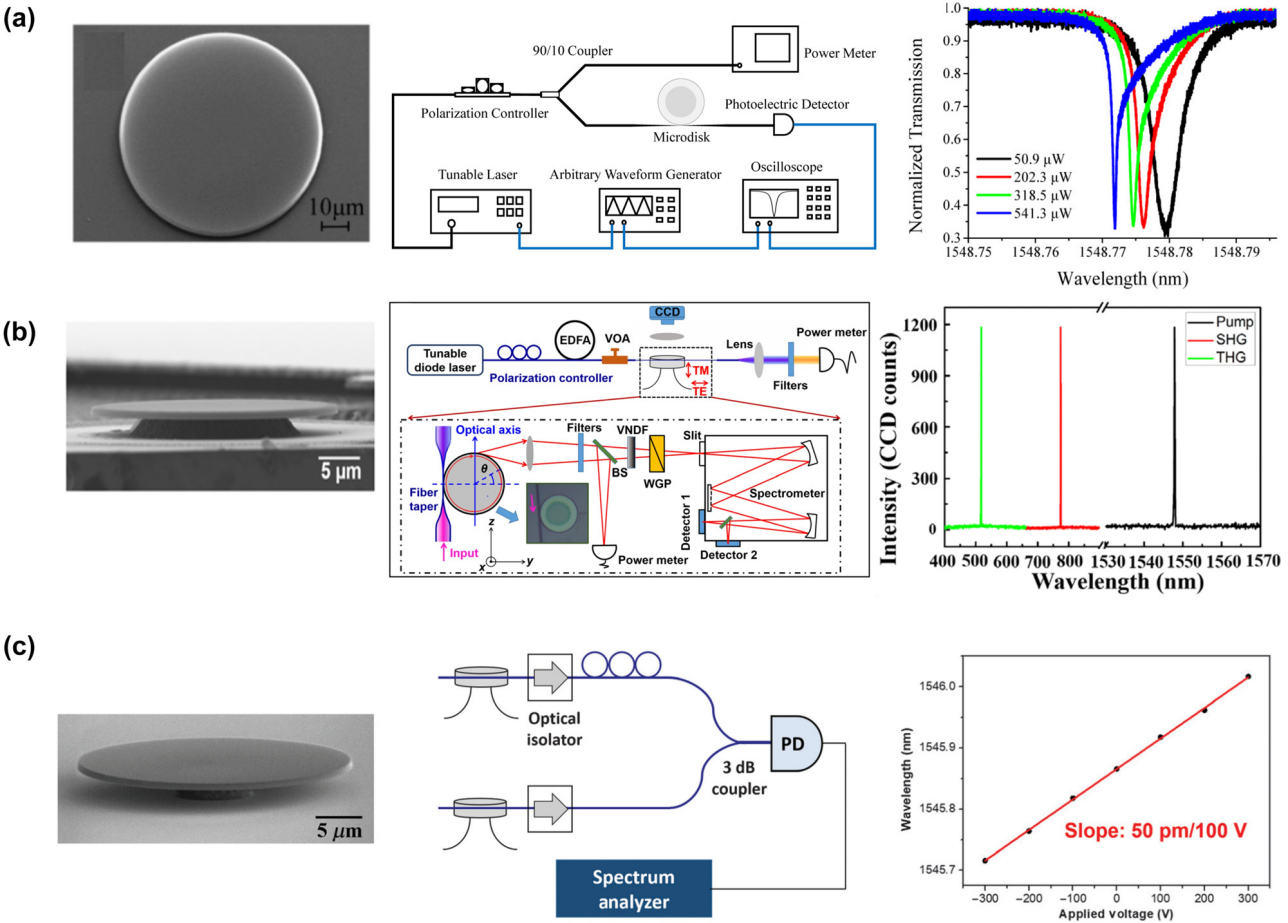
The disc resonator devices based on LNOI. (a) The resonance of an LNOI micro-disk was broadened or compressed by tuning the wavelength of the input laser to the shorter or longer wavelengths [176]. (b) Second- and third-order harmonics were generated in the transverse electric mode propagating along the circumference of the x-cut LNOI micro-disk resonator, where QPM were realized in periodic variation of the TE polarization *d*_eff_. He second-harmonic and cascaded third-harmonic waves are generated simultaneously with normalized conversion efficiencies as high as 9.9 %/mW and 1.05 %/mW^2^ [177]. (c) The EO modulation efficiency of the disc resonator device is up to 50 pm/100 V [178].

In 2016, Xu et al. experimentally observed and theoretically analyzed TO effects in high-Q on-chip LNOI micro-disks. The resonance of a LNOI micro-disk was broadened or compressed when the wavelength of the input laser was tuned to shorter or longer wavelengths (see Figure 7(a)) [176]. Cheng et al. fabricated an x-cut LNOI micro-disk resonator using femtosecond laser writing and wet chemical etching to generate second- and third-order harmonics and realized EO modulation [177], [178]. The second and cascaded third harmonics are generated simultaneously with normalized conversion efficiencies as high as 9.9 %/mW and 1.05 %/mW^2^ (see Figure 7(b)), and the EO modulated efficiencies reach up to 50 pm/100 V (see Figure 7(c)).

**Figure 8:**
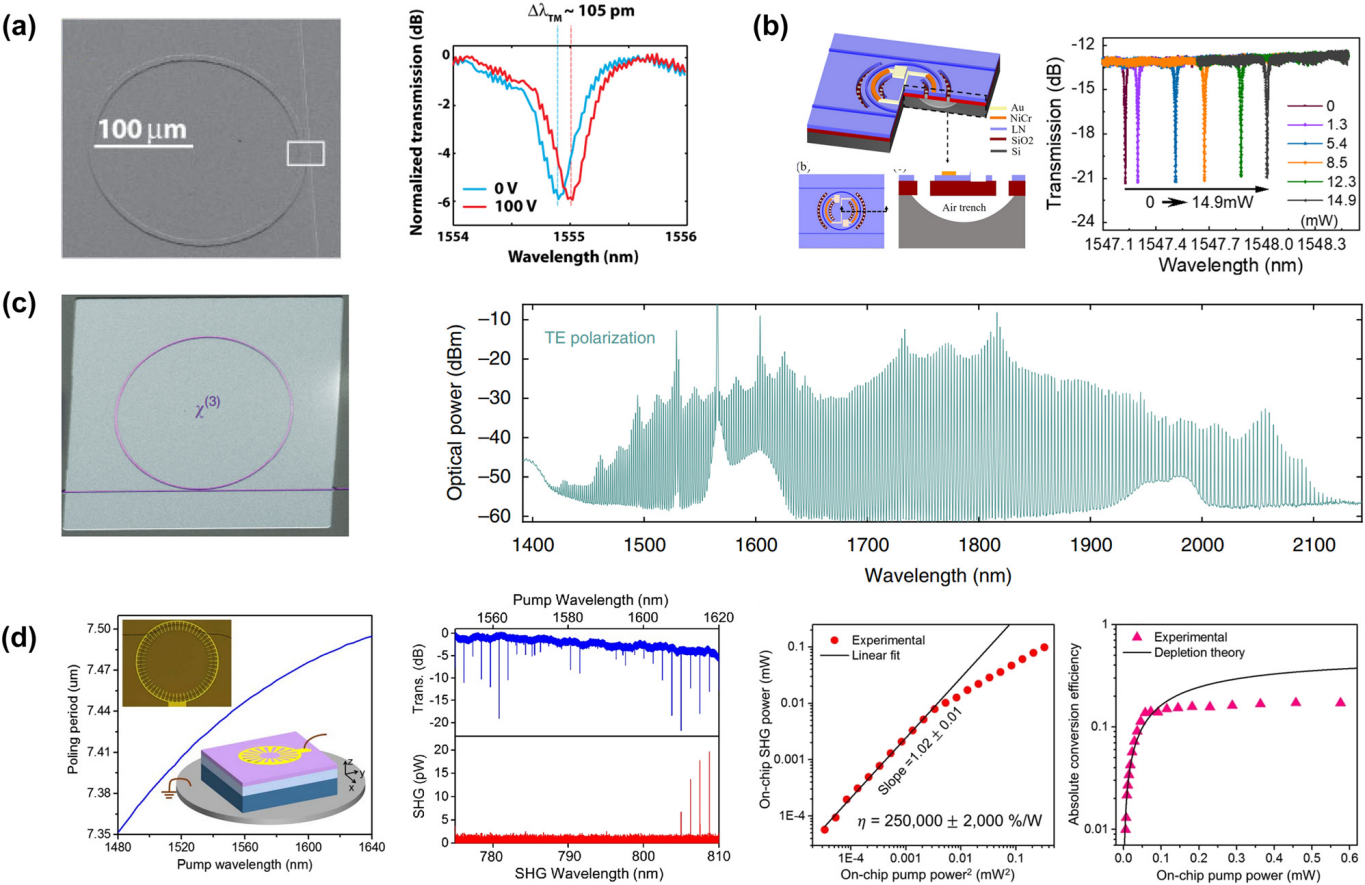
The ring resonator devices based on LNOI. (a) The EO micro-ring resonators shifted 105 pm/100 V [179]. (b) Wavelength shift and FWHM as a function of the heating power of the proposed device as TO tuning performance [180]. (c) High-efficiency SHG were illustrated in dual-resonant, periodically poled z-cut LNOI micro-rings, where QPM is realized by field-assisted domain engineering. An on-chip SHG efficiency of 250,000 %/W is achieved in the low power regime at around 1617 nm and the conversion efficiency of 15 % is recorded when power of 115 μW pump in the waveguide [181]. (d) Electrically programmable add-drop filter modulated Kerr OFC generation on-chip [182].

There has been increasing attention to the EO effect in LNOI since 2007 when Guenter and his colleagues reported the first realization of optical micro-ring resonators in sub-micrometer thin films of LNOI by an improved crystal-ion-slicing and bonding technique using benzo cyclobutene (Figure 8(a)) [179]. The curve shifted by approximately 105 pm when a voltage of *V* = 100 V was applied to the device electrodes at a wavelength of around 1.555 µm. The shift corresponds to an approximate tunability of 0.14 GHz/V. Later, Cai et al. demonstrated a high-efficiency TO tunable micro-ring resonator with a full free spectral range wavelength shift of 14.9 mW heating power in thin-film LNOI (see Figure 8(b)) [180]. Wavelength shift and FWHM can be regarded as functions of the heating power of the proposed device, serving as indicators of the TO modulation power. In 2019, Lončar et al. designed a LNOI-based broadband Kerr OFC generation on a single ring chip, which could be modulated by an electrically programmable add-drop filter (see Figure 8(c)) [181]. The Kerr OFCs were generated with a line spacing of. ∼250 GHz, and span ∼300 nm–700 nm for TM and TE modes, respectively. Q. Lin experimentally reported an LN micro-ring resonator with a high SHG conversion efficiency of 1500 %/W in the 1550- and 780-nm bands through MPM [88]. Soon after, Tang et al. demonstrated SHG in a coupling design and a high-Q LNOI micro-ring, achieving SHG efficiencies of 250,000 %/W in the low-power regime at around 1617 nm, and a conversion efficiency of 15 % at a pump power of 115 μW in the waveguide [182] (see Figure 8(d)).

### 2.4 Metasurface and photonic crystal

Although conventional QPM schemes have been successfully applied, the technique also has its drawbacks. For example, the electro-polarization technique can only be used to generate binary phase states (0 and π) in nonlinear polarization rates. However, this binary phase state may result in some undesired nonlinear optical processes. In addition, since the periodic unit of polarized materials is usually much larger than the wavelength of light, this may generate unwanted diffraction. If the two problems of binary phase and large cell size can be overcome, more efficient and better control of nonlinear optical processes will be possible with the QPM. Differently, the nonlinear metasurfaces allow spatially continuous modulation of polarizability phase (from 0 to 2π), and its emergence provides us with a new solution to the above problems. Metasurfaces have been widely used to simultaneously modulate the linear and nonlinear responses, provided that a suitable subwavelength unit can be selected in the design of nonlinear optical materials. Typically, PM is essential for nonlinear harmonic radiation and four-wave mixing, but the requirement for this condition can be relaxed significantly for metasurfaces because the effective nonlinear optical processes occur only in the subwavelength thickness of the material layer. In this case, the phase matching condition is no longer as important as in conventional nonlinear optical crystals [183]. The nonlinear efficiency of metasurfaces should be determined by both the polarization rate of the cell and the constituent materials. Since the local EM field strength is extremely sensitive to the geometry of the unit. The phase, amplitude, and polarization properties can be effectively manipulated by modulating the local and global symmetry of the structure surface. Therefore, the nonlinear optical response of the metasurfaces can be tuned by designing functional elements with different geometries. Enhancing the local optical field would improve the nonlinear optical efficiency. In addition, the nonlinear effect can also be generated in LNOI photonic crystal, which may bring new possibilities like topological manipulation [184]. The nonlinear metasurfaces provide a new solution to the above problems, and better control of nonlinear optical processes will be achieved.

**Figure 9:**
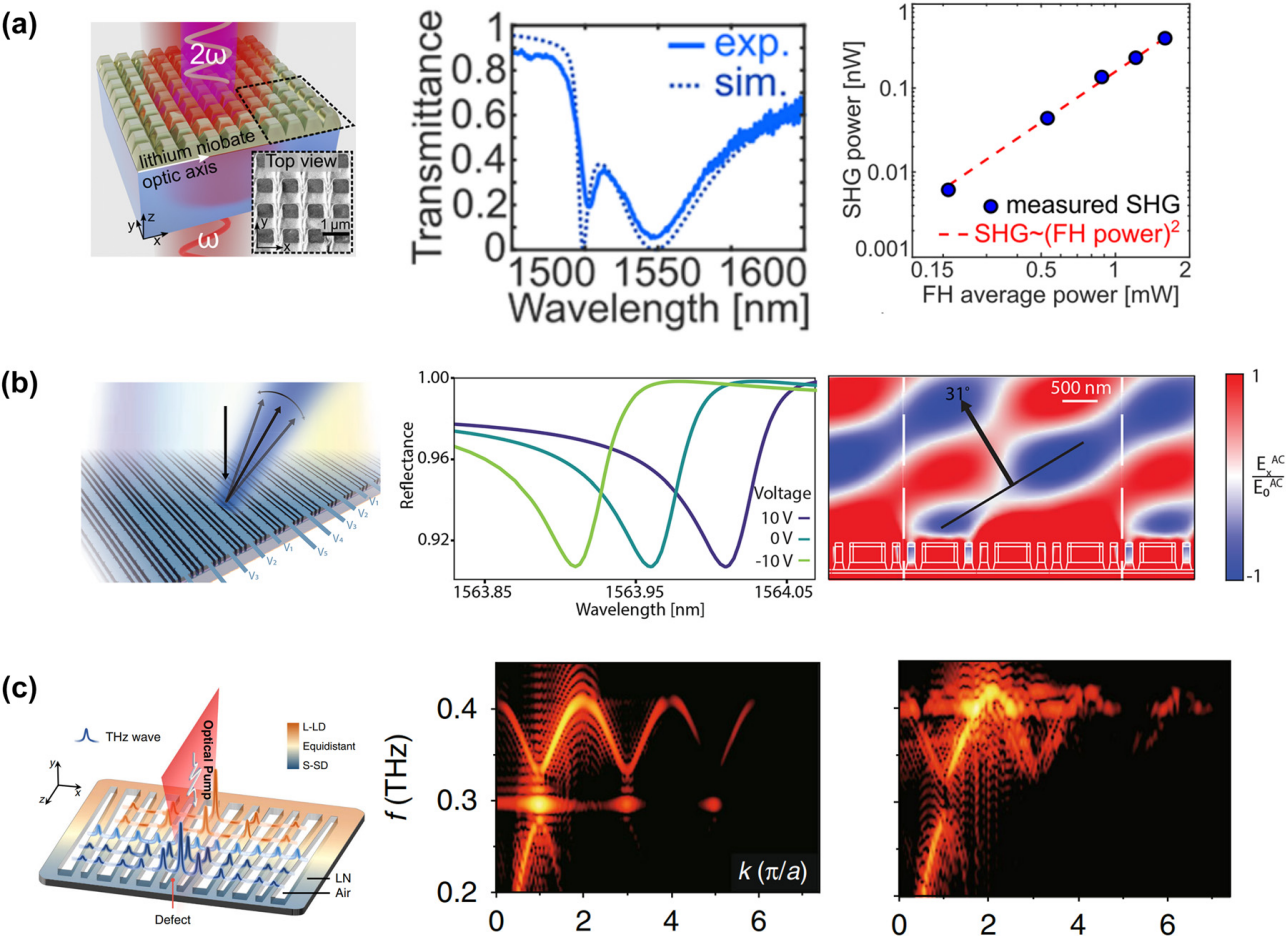
Many applications came true by the nonlinear optical metasurface based on LNOI. (a) The view of SHG in a LNOI metasurface, the fundamental harmonic (FH) is incident from the substrate and the second harmonic (SH) is collected in the forward direction. As seen in the experimental and simulation, SHG power were depend on average power of the FH beam [185]. (b) A high-Q metasurface with full phase tunability with high-angle switchable beam splitter and an angle-tunable beam steerer [186]. (c) Nonlinear generation and topologically tuned confinement of THz waves on a single photonic chip. The nontrivial and trivial defect band-structure were denoted, respectively [192].

In 2020, as shown in Figure 9(a), Setzpfandt et al. fabricated and experimentally studied resonant nonlinear metasurfaces for second-harmonic generation based on thin-film LNOI. Mie-type resonances lead to enhanced SHG in the direction perpendicular to the metasurface [185]. In 2021, the efficient SHG in high Q-factor asymmetric LNOI metasurfaces were reported [186]. Highly efficient SHG were guided to high Q-factor resonances associated with symmetry-protected bound states in the continuum (BIC) in LNOI metasurfaces. High Q-factor resonances enhance the SH conversion process in the LNOI nanostructures. Meanwhile, Celebrano et al. demonstrated the first monolithic nonlinear periodic metasurface operating in the visible range based on LNOI. The efficient steering and polarization encoding were achieved by adjusting the pump polarization [187]. In 2022, Xu et al. experimentally demonstrated a record high SHG efficiency of 2.0 × 10^−4^ using an LN membrane metasurface [188]. The reflex modulators are also exhibited. Levy et al. experimentally demonstrated a new method for free-space rapid optical tunability and modulation. They utilized a planar aluminum nano disk metasurface coated with indium tin oxide (ITO) on a thin film of LNOI with a chromium/gold (Cr/Au) substrate [189]. Light scattering is caused by the complexity, spatial, and temporal inhomogeneity of light propagation paths in a medium. Its manipulations and applications are long-awaited but remain a challenging goal. In this context, Barton and his colleagues have proposed a series of high-Q metasurfaces with full phase tunability, high-angle switchable beam splitter, and an angle-tunable beam steerer [190]. In 2022, as illustrated in Figure 9(b), they designed a metasurface based on LNOI for efficient EO wavefront shaping and modulation. This design achieved nearly 2π phase variation along a very high-angle (51°) switchable beam-splitter and 18–31° angle-tunable beam-steering [191]. Devices based on LNOI photonic crystals have also been widely applied. In 2022, Qin et al. designed a LNOI multimode photonic crystal waveguide with a microstructure serrated array of electrodes, enabling reconfigurable beam-steering through wavefront shaping technology [192]. Almost simultaneously, Chen and his colleagues constructed a wedge-shaped Su–Schrieffer–Heeger lattice [193], which can be utilized for nonlinear generation and topologically tuned confinement of THz waves within an LNOI chip (refer to Figure 9(c)). The interaction between nonlinearity and topology has been expanded to the THz wave regime. In 2019, Xu and his colleagues developed a special preparation and treatment process for LNOI. They utilized a focused high-energy gallium ion beam to selectively remove the LNOI molecules [194]. An array of nanowires arranged in an ordered cycle was processed on the surface to achieve selective transmission of incident light color. In 2023, Lin et al. designed and simulated LNOI nano-ring resonators with reflection peaks in red, green, and blue based on Mie magnetic dipole (MD) and electric dipole (ED) resonances [195].

**Figure 10:**
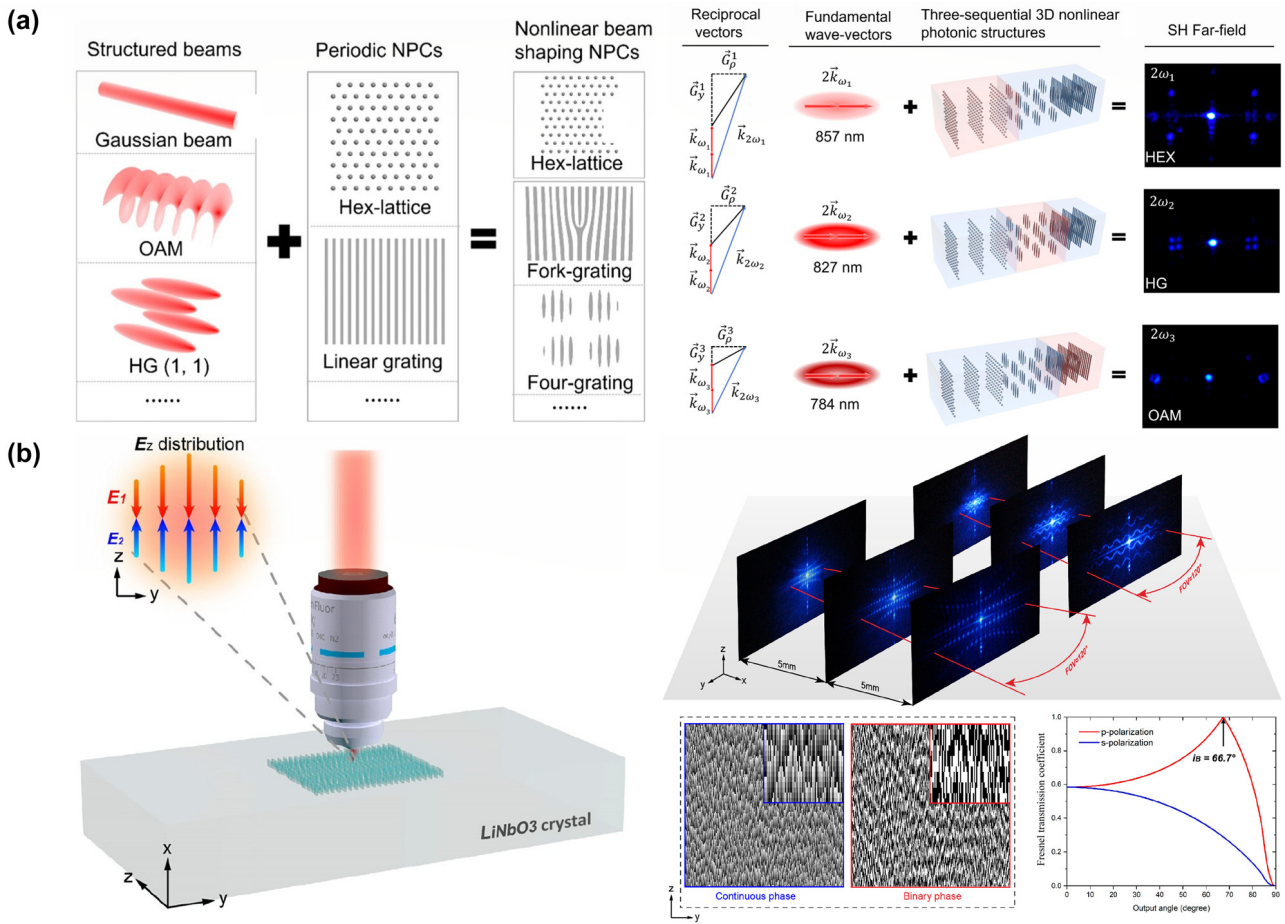
The nonlinear hologram fabricated by using the femtosecond laser writing technique in LN. (a) Schematic diagram of the combination of structured beams and periodic photonics crystals. Based on the sequential design idea, different combinations can realize nonlinear beam shaping in different situations. The SH structured beams are emitted from different action regions in the three-sequential 3D photonics crystals at input wavelengths of 857, 827, and 784 nm, respectively, which means the output SH patterns can be changed by tuning the wavelength of the fundamental wave [196]. (b) The schematic of the laser writing system in LN nonlinear holograms and the SH dots and wavy lines with the phase hologram for the generation of SH wavy lines calculated by using the G–S algorithm. The dependence of Fresnel transmission coefficients at an SH wavelength of 400 nm on the output angle from LN to air is shown. The fundamental wavelength is 800 nm and the SH pattern within an FOV of ∼120° [197].

Nonlinear holograms fabricated by laser writing techniques have attracted a lot of attention for their fascinating functions in laser display, security storage, and image recognition. In 2023, Wu et al. demonstrated a nonlinear hologram fabricated using the femtosecond laser writing technique within an LN crystal [196]. The output direction of the nonlinear beams can be altered by the carrier frequencies, while their efficiencies can be enhanced by the quasi-phase matching condition. In 2024, as seen in Figure 10(b), Zhang et al. experimentally demonstrated an ultra-high-resolution LN hologram using the laser poling technique, achieving a minimal pixel size of 200 nm and extending the field of view (FOV) to over 120° [197]. LN metamaterial fabricated by laser provides a powerful platform for manipulating nonlinear optical interactions for advanced applications across different wavelength bands.

Here, we discuss nonlinear metamaterials in a quantitative manner and compare their performance with that of integrated photonic devices (see Table 1). It should be noted that the nonlinear optical metamaterials reported in the paper have shown very limited performance. This is mainly because metamaterials are very thin, so the weak nonlinear effect is rarely accumulated. Although the high-Q resonance significantly boosts the conversion efficiency due to an increase in interaction time, it results in a narrow bandwidth. So far, the Q-factors of the metamaterials are much smaller than those of the ultra-high-Q ring or disk (micro-)resonators. Additionally, the coupling rate to the high-Q resonance supported by the metamaterials may be limited because of their inherent radiative characteristics. We also introduce some representative EO, AO, and TO modulation efficiencies based on thin-film LN modulators, as summarized in Table 2, including MZI modulators, disk and ring micro-resonators, and metasurface modulators.

**Table 1:** The SHG efficiency of nonlinear photonics devices based on LNOI.

Structure	Method of PM	SHG efficiency (W^−1^)	Ref
Ridge waveguide	BPM	4.7 %	[79]
Ring	MPM	1500 %	[87]
Ridge waveguide	QPM	2600 %	[134]
Disk	QPM	9900 %	[176]
Ring	QPM	250,000 %	[182]
Metasurface	High-Q resonance	10^−6^	[185]
Metasurface	High-Q resonance	2 × 10^−4^	[188]

**Table 2:** The modulation efficiency of photonics devices based on LNOI.

Modulator type	EO/AO/TO modulator	Modulation efficiency	Ref
MZI	EO	*V*_*π*_ = 1.4 V	[136]
MZI	EO	*V*_*π*_ = 5.1 V	[140]
MZI	AO	0.073 rad/ $\sqrt{mW}$	[141]
Disk	EO	3.0 GHz/V	[152]
Disk	EO	50 pm/100 V	[178]
Disk	TO	28.36 pm/mW	[180]
Ring	EO	105 pm/100 V	[185]
Metasurface	EO	6 nm/100 V	[189]

Besides the challenges posed by the thinness of metamaterials, there are limitations of LNOI that need to be overcome, such as DC drift, the photorefractive effect, and limited CMOS compatibility. The DC drift can be controlled by engineering the electrical conductivity of the LNOI substrate [198]. The photorefractive effect can be suppressed through the use of properly ion-milled LN crystals and/or improved removal of photon-excited free electrons by a high electric field [199]. Additionally, it is feasible to construct microstructures on LNOI platforms for photonic circuits, which are also compatible with CMOS technology [17]. For example, ultralow-loss and high-confinement nanophotonic LNOI waveguides and high-Q micro-resonators are fabricated through CMOS-compatible microstructure engineering of LNOI [79], [134], [135], [152], [181], [182]. Also, the MZI based on LNOI has excellent characteristics that are compatible with CMOS technology and is highly regarded for future optical computing chips [113], [136], [140], [200].

## 3 Conclusions and prospective

In this review, the nonlinear physical mechanisms, tuning methods, device configurations, and recent developments are comprehensively discussed. First, the *pros* and *cons* of various approaches for PM as well as their applications, were presented. BPM could be achieved when the birefringence effect precisely offsets the dispersion effect, resulting in low efficiency. In addition, QPM could be achieved by periodically modulating the nonlinear polarization rate. However, this binary phase state cannot allow for spatially continuous modulation of polarizability phase (from 0 to 2π), which limits the application of non-linear optics. The nonlinear metasurfaces offer a new solution to the aforementioned problems, enabling better control over nonlinear optical processes. Currently, LNOI is highly manipulable and is extensively utilized for EO, TO, and AO modulation because of its significant EO, TO and AO modulation due to its large EO, TO, and PO coefficients. In addition, this review also discusses the microstructures of nonlinear optical devices, such as ridge waveguides, periodically poled inversion waveguides, MZI modulators, micro-resonators (disks, rings), metasurfaces, and photonic crystals. Finally, the linear nano-optical application, such as structural colors, is also introduced.

With the development and perfection of integrated photonics chip theory, preparation, and application, LNOI provides strategic basic support for the advancement of integrated photonics. The performance of LNOI has obvious advantages in thin film filters and integrated optoelectronic devices. LNOI is known as the key material for the new generation of information and communication technology. We anticipate breakthroughs in several areas in the coming years. For instance, more complex 2D and 3D structures for LNOI metamaterials and photonic crystals will be developed. The easy doping of LN is also well-suited for generating a gain medium. Laser processing of LNOI is also a very attractive process. We hope that more explorations will be reported in the near future, and that this review will serve as a stimulus for new revolutionary applications in optical communication and quantum technologies.
